# The 'hidden' burden of malaria: cognitive impairment following infection

**DOI:** 10.1186/1475-2875-9-366

**Published:** 2010-12-20

**Authors:** Sumadhya D Fernando, Chaturaka Rodrigo, Senaka Rajapakse

**Affiliations:** 1 Department of Parasitology, Faculty of Medicine, University of Colombo, 25, Kynsey Road, Colombo 8, Sri Lanka; 2 University Medical Unit, National Hospital of Sri Lanka, Colombo, Sri Lanka; 3 Department of Clinical Medicine, Faculty of Medicine, Colombo, Sri Lanka

## Abstract

**Background:**

The burden of post-malaria cognitive impairment is often overlooked. Given the large number of infections occurring worldwide, the magnitude of the problem is likely to be substantial. The objectives of this paper are; (i) to assess the evidence on post malarial cognitive impairment or impact on school education; (ii) to assess the possible positive impact of malaria drug prophylaxis on cognition; and (iii) to suggest recommendations on minimizing the burden of post-malarial cognitive impairment

**Methods:**

PUBMED and SCOPUS were searched for all articles with the key word 'Malaria' in the title field and 'cognitive impairment' in any field. Google Scholar was searched for the same keywords anywhere in the article. The search was restricted to articles published in English within the last 15 years (1995-2010). After filtering of abstracts from the initial search, 44 papers had research evidence on this topic.

**Results & Discussion:**

Cognitive abilities and school performance were shown to be impaired in sub-groups of patients (with either cerebral malaria or uncomplicated malaria) when compared with healthy controls. Studies comparing cognitive functions before and after treatment for acute malarial illness continued to show significantly impaired school performance and cognitive abilities even after recovery. Malaria prophylaxis was shown to improve cognitive function and school performance in clinical trials when compared to placebo groups. The implications of these findings are discussed.

## Background

Mortality and morbidity due to malaria is still substantial in many tropical countries. In 2006, 247 million cases of malaria were estimated, resulting in 881,000 deaths [1]. Of the 109 endemic countries, 30 countries in sub-Saharan Africa and five in Asia accounted for 98% of malaria deaths globally [2]. The financial cost to tackle malaria is staggering. The global estimate of direct losses due to malaria (i.e., the personal and public expenditures to prevent and treat the disease) is USD 12 billion annually. Approximately 35.4 million disability adjusted life years (DALYs) are lost in sub-Saharan Africa alone due to the mortality and morbidity of malaria [2]. The estimates of expenditure to tackle malaria globally in 2009 and 2010 are USD 5.335 billion and 6.180 billion, respectively, and include direct costs for diagnosis, treatment and prevention [2].

There exists an important hidden burden of malaria, namely, that of cognitive impairment and effects on school performance resulting from malaria infection in children. This effect could be postulated to have a long-lasting impact on patients' lives preventing them from achieving their full potential. This impact is difficult to summarize in terms of DALYs or traditional cost evaluation methods of estimating direct losses due to illness. However, given the large number of infections occurring worldwide annually, and the potential impact on lives of patients and family, this hidden burden is likely to be considerable.

A review published by Holding *et al *in 2001 on the same topic concluded that malaria infections (especially severe and cerebral malaria) have a lasting effect on cognition, behaviour and performance in children [3]. However, they also noted that all are not affected to the same extent and these manifestations may be subtle in many children. Many studies on post malarial cognitive impairment have been published since then and our understanding of the burden of post malarial cognitive and behavioural problems has immensely expanded. A more recent review by Kihara *et al *(2006) on post malarial cognitive impairment conclude that a significant proportion of children with cerebral malaria and severe falciparum malaria are at risk of subsequent cognitive impairment. This impairment in cognitive abilities was seen in all cognitive spheres; language, attention, memory, visuospatial skills and executive functions [3,4].

This paper reviews the current evidence on cognitive impairment due to malaria and aims: (i) to assess the evidence on post malarial cognitive impairment or impact on school education; (ii) to assess the possible positive impact of malaria drug prophylaxis on cognition; and (iii) to suggest recommendations on minimizing the burden of post-malarial cognitive impairment.

## Methods

PUBMED and SCOPUS were searched for all articles with the key word 'Malaria' in the title field and 'cognitive impairment' in any field. Google Scholar was searched for the same keywords anywhere in the article (both terms together). The keywords were kept less specific to collect all relevant articles in the first screening. The search was restricted to articles published in English within the last 15 years (1995-2010). There were 971 abstracts in the original search with these restrictions. The software Endnote X3 (Thomson Reuters, Carlsbad, CA 92011, USA) was used to filter articles. Bibliographies of cited literature were also searched. All abstracts were read independently by the three authors, and key articles were identified based on a consensus among all authors. Related or cited papers were also included. Sources were screened for relevance to the topic. These sources included review articles published in core clinical journals, cohort studies, interventional studies, case control studies, case series and cross sectional analyses related to malaria and post infection cognitive impairment. The epidemiological data were downloaded from the websites of international agencies, such as the World Health Organization (WHO). After filtering of abstracts from the initial search, only 44 papers had research evidence on this topic.

### Post malarial cognitive impairment: evidence from studies

In the past, recovery from cerebral malaria has generally been thought to be complete. Early studies suggested that neurological sequelae were relatively uncommon [5]; however findings over the last decade have shown this assumption to be incorrect. Many recent studies have documented significant cognitive impairment following cerebral malaria infections. One of the earliest studies in this regard was by Dugbartey *et al *[6] who assessed somatosensory impairment in 20 Ghanian children after an episode of cerebral malaria and compared them with twenty controls (matched for age and sex). Bimanual tactile roughness discrimination was shown to be impaired in the cerebral malaria group while other somatosensory modalities were intact. While this study did not assess the cognitive functions per se, it paved the way for future studies by establishing evidence for permanent neurological sequelae of cerebral malaria.

Idro *et al *[7] reported severe residual cognitive and neurological impairment in 23 children with a clinical history of cerebral malaria in Uganda. The sequelae of cerebral malaria in this sample included spastic motor weakness (14), loss of speech (14), hearing deficits (9), behavioural problems (11), epilepsy (12), blindness (12) and severe cognitive impairment (9). The sample was from a tertiary care referral centre, and thus the frequency of these deficits was not representative of the population. The children assessed in this sample were in an age range of 12-79 months, and for many, application of formal cognitive testing would have been difficult. As the authors admit, formal IQ testing had not been used to assess the cognitive impairment given the cultural variation in the sample. Instead, cognitive impairment was documented if the child had impaired response to play, communication, safety, self-care and age adjusted school performance. It is unclear how the cognitive impairment was graded as being mild or severe. However, an important finding in this study is the observation of other neurological sequelae such as blindness (observed to be reversible), loss of speech (relatively long-lasting), hearing defects and epilepsy. Such deficits in the early developmental years will undoubtedly impair the child's ability to learn and relate to peers and family, leading to impaired cognitive development.

It is interesting to note the high percentage of behavioural problems observed in this series which included behaviours closely resembling a formal diagnosis of attention deficit hyperactivity disorder (ADHD), conduct disorder and pervasive developmental disorder. While it may be questioned whether the behavioural disorders were causally linked to cerebral malaria, it should be noted that the behavioural problems had started after recovery from cerebral malaria. The changed behaviours were well noticeable and disruptive to daily functioning of the child and family. These changes could be attributed to an alternative explanation (side effects of phenobarbitone) in only one child.

Another study of behavioural problems following a diagnosis of cerebral malaria (n = 64), has also reported effects such as withdrawn/depressed behaviour(15.6%), thought problems (12.5%), aggressive behaviour (9.4%) and oppositional defiant behaviour (9.4%)[8].

Though cerebral malaria may have a causal role in cognitive impairment, some authors hold that seizures per se during a malarial episode may not be strongly associated with cognitive impairment. Kihara *et al *[9] assessed everyday memory in three samples of children following recovery from malaria. The three groups were: patients with cerebral malaria, patients with malaria plus complex seizures and those without any of the above. Everyday memory was assessed with the Kilifi Creek Behavioural Memory Test for Children which assessed the memory required to carry out activities of daily living (recall, recognition and prospective memory). Children with cerebral malaria had significantly low scores in everyday memory compared to those with malaria plus seizures and healthy controls (p = 0.003). The impairment was significant in recall (memory of past events) and recognition (recognition of previous exposures following clues) subcategories. However, in addition to a diagnosis of cerebral malaria, several other demographic and illness related criteria were also associated with impaired memory in univariate analysis. These included poor nutrition, not having schooled, having more than nine seizures and having a seizure duration of more than 30 minutes (p < 0.05). However, on multivariate analysis with logistic regression, only schooling, nutrition and a diagnosis of cerebral malaria were significantly associated with memory impairment (p < 0.05).

In addition to short-term ill effects, cerebral malaria may have a long-term impact on cognition as well. Carter *et al *retrospectively assessed a cohort of children aged 6-9 years who had an episode of cerebral malaria [10-12]. Again the three groups for analysis were similar, namely; children with a past diagnosis of cerebral malaria, malaria with seizures (without coma) and healthy community controls. The follow up was after 20-112 months after the illness. These children were subjected to a battery of testing on areas of cognition, speech and language, motor skills and behaviour. Compared to healthy controls, those with a past history of cerebral malaria showed significantly impaired functioning (p < 0.05) in speech and language tasks (higher level language functions, vocabulary, pragmatics, phonology) and cognition (non verbal functioning). The malaria-with-seizure group also showed impairment in the speech and language domain (syntax, pragmatics, phonology) and in behaviour but not in cognition (attention and non-verbal functioning). Active epilepsy after malaria was associated with significant impairment (p < 0.05) in all domains namely; speech and language, cognition, behaviour and neurological deficits. Though having seizures and their frequency or duration was not significantly associated with cognitive impairment (in multivariate analysis) in the study by Kilhara *et al*, Carter *et al *showed that persistent epilepsy after malaria has an impact on cognition. Abubakar *et al *[13] assessed the cognitive functions of children with cerebral malaria (who were enrolled to a trial that assessed the efficacy of prophylactic phenobarbitone to control the seizures in acute illness) at 3 months and 12 months post discharge. However no difference in cognitive abilities were seen in the treatment arm and the placebo arm indicating that phenobarbitone prophylaxis did not improve cognitive functions in treatment group post-infection. However it was also observed that recurring seizures (in both arms) was associated with subsequent cognitive impairment. While this confirms the findings of Carter *et al*, the role of seizure prophylaxis during an acute phase is still open to discussion. The failure to see a benefit in the treatment arm may be due to the use of phenobarbitone in the initial study which itself can cause cognitive impairment.

In another prospective study over two years, John *et al *[14,15] assessed long-term cognitive impairment in three groups of children with cerebral malaria (n = 44), uncomplicated malaria (n = 54) and healthy community controls (n = 89). The cognitive areas assessed were memory, attention and tactile-kinesthetic learning. At the end of the follow up, a diagnosis of cerebral malaria was significantly associated with impairment of either one or more cognitive domains (compared to uncomplicated malaria group and normal controls) (p = 0.006). Interestingly, the deficits were most seen in the attention domain. After multiple linear regression to adjust for confounding factors, a diagnosis of cerebral malaria carried a relative risk of 3.67 for cognitive impairment compared to healthy children. Three other studies in Mali, Senegal and Kenya have also shown that a past history of cerebral malaria is associated with impaired cognition, putting the victims at increased risk of intellectual impairment [16-18].

Many authors have acknowledged the difficulty in applying formal neuropsychological testing adopted in western populations to rural African communities given the cultural, language and educational differences. Therefore Kihara *et al *[19] have recently attempted to use an 'objective' independent measure of cognitive impairment that is not affected by these phenomena. They assessed the event related potentials (ERP) of cerebral activity of healthy children exposed to novel or unexpected stimuli and compared with the same of children affected by severe malaria (cerebral malaria, malaria with seizures and malaria with prostration). Both groups were exposed to novel visual and auditory stimuli and had their ERPs recorded. Up to 14% of children who has had severe malaria showed impaired ERP recordings indicating that a significant proportion of children exposed to severe malaria have impaired neuro-electrical responses to novel stimuli.

Reliable predictors for cognitive impairment following cerebral malaria are clearly of importance. Kihara *et al *[9] identifies multiple episodes of hypoglycaemia and profound coma (p < 0.05) (but not seizures) to be predictors of later cognitive impairment. Carter *et al *[12] in their study have shown that neurological status at discharge is not predictive of later neurocognitive impairment. However they have demonstrated that active epilepsy following infection is associated with significant cognitive impairment [10]. Holding *et al *[17] suggest that a combination of four features, namely: seizures, hypoglycaemia, coma and absence of hyper-pyrexia  have a better predictive value of subsequent cognitive impairment than the presence of gross neurological signs at discharge. Idro *et al *[20] in a separate study analyzed the hospital records of 143 children aged 6-9 years diagnosed with cerebral malaria and showed that 24% of this sample had impairment in one or more domains of motor, speech and language and cognitive areas. Profound coma, severe malnutrition and hypoglycaemia during the acute episode were associated with cognitive impairment while malnutrition, age less than three years, hypoglycaemia and evidence of increased intracranial pressure were associated with speech and language disorders. Seizures were only associated with motor impairment. Boivin *et al *[15]describe that cognitive impairment at six months of follow up is a strong predictor of a long-term cognitive impairment. They also report that duration of the coma and the number of seizures before treatment are predictors of future cognitive impairment. Birbeck *et al *[21] in their recent publication of the findings of the Blantyre malaria project epilepsy study have found that hyperpyrexia and seizures in acute stage increase the risk for subsequent epilepsy. If this is the case, then seizures in the acute stage may also be indirectly related to cognitive impairment as epilepsy following cerebral malaria is already shown to be associated with such impairment [10,12].

There are few studies assessing the impact of post-malarial cognitive impairment outside Africa. Fernando *et al*, have assessed the impact of malaria on cognitive performance in Sri Lanka where a majority of the malaria infections acquired by children are acute uncomplicated febrile episodes from which they make an apparent complete recovery when treated. Several studies were carried out taking into consideration that school performance is a reflection of the cognitive ability of a child and is dependent on a number of factors including facilities available at school, the quality of teaching and other social factors such as parental supervision. In the first study, Fernando *et al *[22] assessed the impact of malaria on the educational performance at school entry among of 325 children aged 5-6 years. This was a cross sectional survey carried out in two moderately malaria-endemic districts in Sri Lanka. The students were assessed with a standardized entry performance test developed by the National Institute of Education (NIE) of Sri Lanka. It assessed writing skills, language and mathematical performance. Functioning in all domains was poorer in children who have had a life-time rate of more than five attacks of malaria than others (those who had not suffered from malaria and those with a history of less malarial episodes). In a multiple regression analysis, impairment in letter reading was significantly associated with the number of malaria infections after adjusting for other confounding factors (parent's educational status, nutritional status and monthly family income). Although the number of malaria infections experienced by the child could not be confirmed in this study, the trends reported suggested a significant impact of malaria on cognitive performance even at school entry, thus handicapping children who experienced malaria attacks even prior to commencing formal schooling.

In order to confirm these findings, two other studies were carried out in Sri Lanka [23,24]. The impact of repeated attacks of malaria on educational attainment was investigated using a historical cohort where malarial infections were monitored in 571 children resident in a malaria endemic area of Southern Sri Lanka over a six year period. Validated special examination papers were prepared with the aid of the NIE and the results of the school examinations were also considered. Malaria infections were a major predictor of children's school performance in both language and mathematics after controlling for potential confounders (parents education, monthly family income and house type). When all these variables were controlled for, children who experienced less than three attacks of malaria scored at least 15% more in both the special and school examinations than children who experienced more than five attacks of malaria during the same period.

A further investigation was carried out to determine the impact of an acute attack of malaria on short-term educational achievements and on school absenteeism [23]. Six hundred and forty eight children (aged 6-11) were assessed in three categories; children with malaria, children with non-malarial fever and healthy controls. At the time of presentation, the performance in language and mathematics (assessed with age appropriate examination papers) was significantly less for children with malaria compared to those in other categories. When they were reassessed two weeks later, the scores had improved but was still significantly less in the group that had malaria (p < 0.001). This study also demonstrated that absenteeism due to malaria (and not due to non-malarial fevers) is a significant predictor of cognitive performance emphasizing the contention that malaria per se is responsible for the poor school performance. The malaria infections recorded in this study were uncomplicated with the duration of each attack not exceeding 3-5 days as infected children were effectively treated early due to diagnosis and treatment facilities being available through an extensive health care delivery infrastructure. Hence, the impact of malaria on educational performance assumes an even greater importance as such uncomplicated malarial infections occur frequently and repeatedly in children in most malaria endemic regions and are generally forgotten after the event. This is in marked contrast to the rarer form of severe malaria which, in survivors may leave residual effects which are often of a neurological nature.

Al Serouri *et al *[25] in a case control study in Yemen showed that children with parasitaemia performed poorly on formal cognitive testing compared to those without parasitaemia after adjusting for confounding factors. However, a reassessment in two weeks time after therapy showed that elimination of parasitaemia did not result in a significant improvement of cognitive functions. While the follow up period in this study may be short, a similar study in the Brazilian Amazon where a cohort of 198 children (aged 5-14) were followed up for a period of nine months has raised similar concerns [26]. The number of malarial attacks in this cohort was recorded during the follow up and school performance in language and mathematics was assessed by standardized age appropriate examinations at the end of follow up. On a multivariate model, after adjusting for age, mother's level of education, school absenteeism and duration of residence in the study area, having at least one attack of malaria was significantly associated with poor (below the 50^th ^centile for class/grade) school performance (p=0.04). Interestingly, in a small scale study (n=97) Boivin *et al *[27] have demonstrated that though treating asymptomatic malaria parasitaemia did not show an improvement in cognitive functioning (assessed by the Kaufman Assessment Battery for Children), treating chronic intestinal parasitaemias did.

Many of the studies carried out outside Africa have demonstrated that uncomplicated malaria per se was a significant predictor of cognitive function after controlling for potential confounding factors. This further strengthens the fact that malaria contributes significantly to loss of school time, ill health and poor performance.

Many of the studies cited above had arms with children diagnosed as having cerebral malaria. How certainly a diagnosis of cerebral malaria can be made would also affect the interpretation of results. In an autopsy study of 31 children thought to have died from cerebral malaria, Taylor *et al *[28] demonstrates that only 75% of them actually had pathological evidence of death attributable to cerebral malaria. They also observed that changes in the optic fundus during life were more predictive of actually having cerebral malaria than the clinical picture. Such changes in the retina are now referred to as malarial retinopathy. These retinal changes were described by Lewallen *et al *[29] and over the years, the specificity of malarial retinopathy in diagnosis of cerebral malaria has been confirmed in various studies and case series [30-32]. The identified features of malarial retinopathy include; macular and peripheral whitening, vessel whitening including capillary whitening, retinal haemorrhages (predominantly white centered) and papilloedema. The first two changes are so far only observed in malaria. Collectively, malarial retinopathy is shown to have a sensitivity of 95% and a specificity of 90% to diagnose cerebral malaria (as opposed to the specificity of 61% for diagnosing cerebral malaria with WHO specified clinical and laboratory criteria) [28]. It is noted that none of the studies mentioned previously used malarial retinopathy as a diagnostic criteria to define cerebral malaria and therefore some of these children would have had alternate pathologies for clinical picture, outcome and deaths.

Taking this factor into account, Birbeck *et al *[21] followed up 132 children with retinopathy positive cerebral malaria for a median duration of 495 days (compared to a control group without coma or central nervous system infection). Nine percent and 21% of the children with cerebral malaria developed epilepsy and new neurological deficits (gross motor, sensory and language) during follow up and this incidence was significantly more compared to controls. Boivin *et al *[33] in another study assessed the development outcome of eighty three children selected from the same cohort. These children who had retinopathy confirmed cerebral malaria were compared to 95 controls in areas of childhood development [gross motor, fine motor, language and social skills as assessed by Malawi Developmental Assessment Tool (MDAT)] and behavioural problems [assessed by Achenbach Child Behavior Checklist (CBCL; 1 1/2-5 yrs)]. Those who had retinopathy confirmed cerebral malaria showed overall poor scores in developmental assessment with the language domain being the most affected. CBCL scores did not differ significantly between the groups. Platelet and lactate levels at the time of admission was significantly predictive of the MDAT total score, fine motor and language scores while duration of coma was predictive of gross motor and social skill scores.

However, it needs to be appreciated that the studies cited above are not comparable to each other in their designs and results for many reasons. Some have followed up patients with severe malaria, some with cerebral malaria and some have compared these two entities with healthy community controls. Others are more different in that they have enrolled participants from communities who would have had a spectrum of malaria infections from asymptomatic parasitaemia to severe malaria. Similarly, the age range of children tested also varied significantly and this brings forth two issues; the inapplicability of formal cognitive testing to very young children and the differences of cognitive impairments that would be observed depending on the age. The developing brain is more vulnerable to insults but some authors suggest that it may recover from it also due to plasticity. Damage to neuronal circuitry during early childhood may cause obvious defects in memory, language and learning but more subtle changes involving social sensitivity, social integration and executive functions may appear later as the child ages and when such skills become important. On the same note, there are many other confounding factors that can affect the observations of cognitive impairment such as, home and family environment, nutrition, quality of teaching, cultural and language barriers in applicability of neurocognitive testing. In fact, malaria transmission itself is determined by prevalent socioecomonic conditions of the community which may render some children more vulnerable than others and some communities more at risk compared to others. Therefore when referring to cognitive and behavioural effects of the studies cited above, all these coviariates and confounding factors have to be acknowledged. The 'cognitive' impact itself is not a single entity but varies according to the severity of illness, age of the child and methods of measurement.

While there are many studies assessing the cognitive impairment of children after malaria, the impact on adults is not addressed by systematic research. In an earlier study, Dugbartey *et al *[34] have shown that there were no significant neurocognitive deficits in adults after an attack of falciparum malaria compared to healthy controls. The adults recruited in to the study had an age range between 18-60 years and all people with a history of falciparum malaria had their last malarial episode one year prior to the study. The cognitive evaluation was done with objective assessment tools such as mini mental state examination (MMSE), Neuropsychological status examination and SCL-90-R which is a validated self report symptom inventory. However several limitations in the interpretation of these results should be noted. The controls are far fewer than cases in this study (142 vs. 30) and there was no indication of the severity of the initial falciparum malaria infection. Similarly, while tests such as MMSE may pick up gross cognitive impairment it may not be sensitive to subtle changes. Further studies are needed to clarify the impact of malarial infections on the cognition of adults.

### Malaria and cognitive impairment: evidence for causality

It is of fundamental interest to establish whether malaria is causally related to cognitive impairment, poor school performance and other behavioural disturbances as reported in previous studies. Cognitive impairment can be hypothesized to be an end result of many confounding factors (nutrition, other parasitic infections, poor parental care and not having access to proper education) and it is important to understand whether malaria itself can be demonstrated as an independent causative factor for cognitive impairment.

In the study by Idro *et al *[7] the neurological sequelae such as hearing defects, seizures and blindness are clearly documented to occur after cerebral malaria and there is no other alternative explanation for these manifestations. Similarly, the behavioural problems observed in children after the malarial episode were not seen before falling ill. While these neurological deficits and behavioural problems are not synonymous with cognitive impairment, they can indeed retard a child's ability to learn and develop.

In the study by Kihara *et al *[9] which showed certain components of everyday memory to be impaired in children with cerebral malaria, the authors state that a diagnosis of cerebral malaria was made after excluding other causes of encephalopathies. It is not clear how the other encephalopathies were excluded. At the same time on multivariate analysis cerebral malaria was just one factor associated with memory impairment (others being schooling and nutrition). In the studies by Carter *et al *[10,11] the results were adjusted for covariates and confounding factors (age, sex, nutrition, schooling and socioeconomic status) and still significant differences were seen in the cerebral malaria group and those having active epilepsy after malaria compared to healthy controls. Similarly John *et al *[14] also shows a significant risk of cognitive impairment after adjusting for similar confounding factors. In the prospective study by Boivin *et al *[15] which demonstrates cerebral malaria to have significant sequelae of cognitive impairment, the methodology was carefully designed to exclude children with a history of any central nervous system infection (including cerebral malaria), history of chronic illness, previous admissions due to malnutrition and a history of developmental delay. At the same time adjustments were made for confounding factors as described in previous studies.

In the other community based studies outside Africa, similar adjustments were made for confounding factors and still the results showed a significant association even between uncomplicated malaria and cognitive impairment or school performance [22-26].

Overall, in summation, it is fair to assume from reported methodology that all the key studies had made adjustments for potential confounding factors and still found a significant association for post malarial cognitive impairment after complicated/cerebral malaria as well as after uncomplicated attacks. This strongly favours an argument for malaria as an independent causative factor for cognitive impairment. The studies on the impact of malarial prophylaxis on cognition quoted later under the subtopic 'malaria and cognitive impairment; strategies on prevention and rehabilitation' of this article (see below) provides further evidence regarding the causal role of malaria in cognitive impairment. Still, what is obscure is the exact pathology for this impairment. The following section summarizes the current knowledge base in this regard.

### Pathological effects of cerebral malaria and their relationship to cognitive impairment

The pathology of cerebral malaria and its neurocognitive sequelae is still open to debate. It is assumed that parasitic sequestration within cerebral circulation with resultant hypoxia, host immune response and cytokine induced damage via nitric oxide production may contribute to neuronal cell death [35]. Animal studies have demonstrated increased neuronal apoptosis with cerebral malaria [36,37]. John *et al *assessed the levels of different cytokines in cerebrospinal fluid (CSF) in 76 children with cerebral malaria and compared it with eight controls [38]. Many cytokines were raised in CSF in children with cerebral malaria compared to controls (TNF - α, G-CSF, IL-6, IL-8). However, only raised TNF-alpha in CSF at admission showed a positive correlation between impaired neurological function three months later. Raised TNF-alpha was also shown to be correlated with impaired cognition six months later in domains of attention and working memory.

As certain cognitive domains (memory, attention) and higher functions (speech and language) are shown to be selectively affected in cerebral malaria, it is also plausible that imaging studies such as magnetic resonance imaging (MRI) and positron emission tomography (PET) may reveal the involvement of specific brain areas related to these functions to be affected by cerebral malaria. However there are no case series or systematic studies with MRI/PET evidence other than isolated case reports [39,40]. Potchen *et al *[41] report a case series of computed tomography (CT) scan findings of children with cerebral malaria. The acute changes included oedema of thalamic grey matter, cerebral oedema with surrounding infarctions and diffuse cerebral oedema. However these changes were not uniform and only 1-2 children showed changes in each category. Follow up scans in survivors with neurocognitive defects (n=11) had showed local cerebral atrophy of areas in brain initially affected by focal seizure activity during the acute stage. It can be hypothesized that cognitive impairment in cerebral malaria may occur due to damage to frontal lobes (associated with executive functions and retrieval of information), areas of medial temporal lobes and the hippocampal system (involved in memory formation) [9,14,39].

Attempts are being made to model the impact of cerebral malaria in animal models in view of extrapolating the findings to humans. However, such comparisons may not be valid due to species differences of both the host and the parasite. Still, it is observed that mice strain C57BL/6 infected with *Plasmodium berghei *strain ANKA do manifest many characteristics of infection similar to cerebral malaria in humans. Desruisseaux *et al *[42] studied the cognitive impairment following post malaria infection in this host-parasite combination and tried to correlate it with post mortem histopathological findings of the hosts. The visual memory of infected mice was significantly impaired seven days post infection. On histological analysis, margination of inflammatory cells and extravasations of red blood cells were seen in many areas including the thalamus, midbrain and cerebellum. Immunostaining showed significant microglial activation (marker of inflammation) in key areas such as the cerebral cortex and hippocampus. It is hypothesized that extensive microglial activation and inflammation of parenchyma in areas such as hippocampus and posterior parahippocampal region might contribute to the cognitive impairment. The persistence of cognitive impairment despite successful elimination of parasitaemia (by chloroquine therapy) has also been demonstrated in the same animal model[43].

Failure to understand the pathogenesis of cerebral malaria has significantly slowed the development of neuroprotective pharmacological measures during the acute stage of illness. Different agents are being assessed on animal models with promising results but human trials are not still under way. The B5 complex provitamin pantetheine and the immunomodulatory agent glatiramer acetate have both shown promise in animal studies by down regulating the immune response [44]. High dose erythropoietin is another agent that is under investigation for possible neuro-protective effects in cerebral malaria and a recent phase I trial has demonstrated its safety in children with cerebral malaria [45].

### Malaria and cognitive impairment: strategies on prevention and rehabilitation

Cognitive impairment of children living in endemic areas and the impact of prophylaxis has been assessed by a limited number of studies [3,46,47,46,47]. In a double-blind placebo controlled randomized trial, Fernando *et al*[47], showed that educational attainment improved and school absenteeism reduced significantly (p < 0.0001) in children (aged 6-12) taking chloroquine prophylaxis compared with those in the placebo group. This improvement was not associated with any other health-related cause (or non-health issues) and the incidence of malarial attacks in the treated group declined by 55% during the intervention.

A study with a different experimental design [46] assessed the impact of prevention of early childhood malaria on long term cognitive development. The authors assessed cognitive functions in children who participated in a malaria chemoprophylaxis trial 14-16 years previously by Greenwood *et al *[48]. In the initial trial carried out in 1988, children aged between 3-59 months were randomized and offered dapsone-pyrimethamine prophylaxis during the malarial transmission season over three successive years. After the trial, all children less than five years of age were offered prophylactic treatment. These children were re-assessed in 2001 by Jukes *et al *[46] when their mean age was 17 years. Educational attainment was better in the group which had received prophylactic treatment compared with the placebo control, but the scores for the cognitive tests were not significantly different between the prophylactic and placebo groups. The offering of prophylaxis to all children at the end of the trial may have had an impact on equalizing the cognitive scores 14 years later. In a separate analysis, it was shown that the longer an individual had spent in the trial, the better his/her mental development scores were, suggesting a positive impact of malaria prophylaxis on long term cognitive development [46].

A more recent trial by Clarke *et al *[49] assessed the impact of intermittent preventive therapy (sulphadoxine-pyrimethamine in combination with amodiaquine or dual placebo, three treatments over 4 monthly intervals) on improving educational achievement and classroom attention of children aged 5-18 years. Compared to trials mentioned previously, this was a large trial (n = 3535 in treatment arm, n = 3223 in placebo arm) of a stratified, cluster-randomized, double-blinded, placebo-controlled design. Improvements in outcome indicators were assessed 12 months post treatment. Cognitive abilities were assessed by two tests of sustained attention, classroom behaviour was assessed with standardized teacher rated scales and school performance was assessed by a standardized age appropriate examination papers at the end of the observation period. In an intention to treat analysis (75% and 86% of students in each arm had completed at least 7 days of treatment in treatment and placebo groups respectively), the treatment group showed significant improvement in sustained attention tests compared to the placebo group. However there was no statistically significant difference in the teacher-rated behaviour scores for inattentive and hyperactive-compulsive behaviours and educational attainment.

Considering the outcome of this study, a follow up controlled randomized trial is now underway in Kenya that plans to assess the impact of improved health (intermittent screening for malaria and treatment) on improved literacy skills. The randomized design has divided schools in the region to 4 groups (25 schools in each group) to receive malaria intervention alone, literacy intervention alone (skill development of teachers in teaching English and Swahili), both interventions and none (control). The study is expected to be completed by 2012 [50].

Still, rather than concentrating on pharmacological prophylaxis alone, there are many other cost effective simple measures of preventing malaria such as residual insecticide spraying and insecticide impregnated bed nets [51]. Simply put, if the number of infections is reduced, the overall burden of post infective cognitive impairment is also less. Insecticide impregnated bed nets are a low cost socially acceptable strategy that have reduced the incidence of malaria and its associated morbidity in many endemic areas [52,53]. With current impregnation techniques, the insecticide effect can last up to 4 years and this gives a major advantage in prevention strategies.

Another avenue to explore is the prevention of identified adverse prognostic indicators for subsequent cognitive impairment by the studies cited above. These include; preventing hyperpyrexia, hypoglycaemia, malnutrition and seizures during acute illness. The place for such measures needs to be properly assessed by trials.

The 'rehabilitation' strategies for post malarial cognitive impairment are another area assessed by several recent trials. The effect of cognitive training on children affected by severe malaria has shown a positive impact in a study by Bangirana *et al *[54]. Sixty five children diagnosed with cerebral malaria were randomized in to two groups and the intervention group received an eight week training of computer based cognitive skill development tasks. Pre and post intervention cognitive scores were assessed by a computerized neuro-cognitive test battery. The pre treatment scores were similar in both groups. After adjusting for covariates (age, sex, home environment and weight) the group who had cognitive training showed significantly better scores in areas of visuo-spatial processing speed, working memory, learning tasks, psychomotor speed and internalizing problems.

Cognitive rehabilitation in cerebral malaria has still not been evaluated extensively by systematic studies. Much of the speculated benefit of cognitive training is extrapolated from the studies on cognitive training in traumatic brain injury. Though 'traditional' therapist centered or computer based cognitive retraining may indeed be beneficial, the feasibility of such interventions in resource limited settings (lack of trained staff, language restrictions, lack of equipment and unfamiliarity with technology) is questionable. In a recent review on cognitive rehabilitation of children with cerebral malaria, Bangirana *et al *[55] describes the place of standard or computer based cognitive rehabilitation, environmental enrichment, nutritional improvement, speech and physiotherapy in children with post malarial cognitive impairment. However apart from computer based cognitive rehabilitation, evidence for other measures in post malarial cognitive impairment is yet to be established.

### Conclusion and recommendations

The number of studies assessing the impact of malaria on cognitive functions were few and those assessing the impact of prophylaxis were even fewer. The number of patients enrolled in observational studies and case series were in the range of 20-200 except in the trial by Clarke *et al *[49]. In addition, all these studies have enrolled either children or adolescents and none have assessed the cognitive impairment in adults due to either uncomplicated or cerebral malaria. Most of the studies were conducted in Kenya, Africa and in other African populations with only a few studies from the rest of the world. The methods of assessment of cognitive functions also varied with the different types of formal tests used and the cognitive domains tested. In addition, some authors have assessed school performance directly rather than the cognitive domains themselves. However, these studies were included in this review as it is an indirect yet more pragmatic measure of cognitive functions and its deterioration following malaria.

However despite these limitations some common themes emerge in the analysis of observations in the previously mentioned studies:

1. The cognitive abilities and school performance were impaired in the groups with malaria (either cerebral malaria or uncomplicated malaria) in almost all the studies at a statistically significant level compared to healthy controls.

2. Hypoglycaemia, seizures and coma during cerebral malaria have been shown to predict later cognitive impairment [9,17,20].

3. While observations are made regarding cognitive impairment based on neuropsychological test batteries that concentrate on clinical performance, these are not supported by biochemical or imaging studies which could help to elicit the underlying pathology.

4. Studies comparing cognitive functions before and after treatment of acute illness continue to show significantly impaired school performance and cognitive abilities even after recovery compared to healthy controls [23,25,26].

5. However, the three studies on malarial prophylaxis all show improved cognitive function/school performance compared to placebo groups at the end of follow up which in two studies were more than one year [46,47,49].

Given the large number of infections in endemic areas, these findings carry much weight and needs to be highlighted. This 'hidden' burden of malaria is often unnoticed and overlooked but may place vulnerable populations (especially children) at a disadvantage by causing cognitive impairment resulting in poor school performance and non achievement of full potential in life. This burden of illness is difficult to quantify and is probably much larger than the already publicized tangible costs of malaria. It must also be highlighted that treating an established attack of malaria in a child and preventing malaria in the same child have different consequences. As suggested by the available evidence, the latter option is the better choice.

Therefore it is recommended that:

1. Countries where malaria is endemic should pay special attention to prevent malaria by appropriate prophylaxis for populations at risk.

2. Such programmes must pay special attention to infants, toddlers and children as this is a crucial age in cognitive development and for schooling. However, cognitive impairment in adults may also be significant but unfortunately this has not yet been assessed properly.

3. The impact of malaria on cognitive development must be highlighted and the public should be educated to ensure compliance in prophylaxis campaigns.

4. Early identification and treatment of malarial fever should be given priority with providing necessary infrastructure and staff education on clinical and parasitological diagnosis.

5. Attacks of cerebral malaria, coma, seizures and hypoglycaemia during an acute attack must be actively looked for and avoided to prevent later cognitive impairment and local guidelines should stress these facts.

Areas for further research on this timely topic include;

1. Establishing the effect of acute malarial infections and prophylaxis on cognitive functions of adults in endemic areas.

2. Observational studies to identify the pathophysiology of post malarial cognitive impairment by correlating biochemical markers, serum and CSF cytokine levels and imaging studies with clinical findings.

3. Assessment of long term cognitive impact of recurrent malarial attacks with a longer prospective follow up (more than one year) with intermittent cross sectional assessments.

4. Development of methods to quantify the impact of post malarial cognitive impairment in terms of financial costs.

## Competing interests

The authors declare that they have no competing interests.

## Authors' contributions

All authors have participated in designing, article search, information coding and writing of the manuscript. All authors have read and approved the final manuscript
